# A Village in the Southeastern Region of Iran Harboring the c.716T>A (p.Val239Asp) Mutation in *SLC26A4*

**DOI:** 10.34172/aim.28745

**Published:** 2024-09-01

**Authors:** Farzane Zare Ashrafi, Saeed Dorgaleleh, Raziye Rezvani Rezvandeh, Negar Kazemi, Nasrin Azizi, Masoud Edizadeh, Mohammad Hossein Azizi, Kimia Kahrizi, Hossein Najmabadi, Reza Najafipour, Marzieh Mohseni

**Affiliations:** ^1^Genetics Research Center, University of Social Welfare and Rehabilitation Sciences, Tehran, Iran; ^2^Department of Bioinformatics, Genoks Genetic Diagnosis Center, Ankara, Turkey; ^3^Ilyome Bioinformatics, Ankara, Turkey; ^4^Academy of Medical Sciences of IR Iran, Tehran, Iran

**Keywords:** Exome sequencing, Founder mutation, Iran, Non-syndromic hearing loss, *SLC26A4*

## Abstract

After *GJB2, SLC26A4* is the second most common contributor to autosomal recessive nonsyndromic hearing loss (ARNSHL) worldwide. In this study, we used Exome Sequencing (ES) to present a village with 31 individuals affected by hereditary hearing loss (HHL) in southeastern Iran near the border of Pakistan. The village harbored the known pathogenic missense *SLC26A4* (NM_000441.2):c.716T>A (p.Val239Asp) mutation, which has a founder effect attributed to Pakistan, Iran’s southeastern neighbor. Our findings, in addition to unraveling the molecular cause of non-syndromic hearing loss in these patients and further confirming the common ancestry and migration story between the people of this region and Pakistan, provide further insight into the genetic background of this region and highlight the importance of understanding the mutation spectrum of *GJB2* and *SLC26A4* in different regions to choose cost-effective strategies for molecular genetic testing.

## Introduction

 Hereditary hearing loss (HHL) is the second most prevalent clinical and genetically heterogeneous neurosensory disorder and health concern in Iran.^1^ HHL affects 1/166 Iranian individuals due to the high rate of consanguineous marriages in Iran.^1^ According to comprehensive studies of the Genetics Research Center (GRC) of the University of Social Welfare and Rehabilitation Sciences (USWR), Tehran, Iran, over two decades, the six most prevalent genes mutated in non-syndromic hearing loss (NSHL) Iranian patients were *GJB2*, *SLC26A4*, *MYO15A*, *MYO7A*, *CDH23*, and *TMC1.*^1^ *SLC26A4 *(MIM:605646) mutations are assumed to be the second most frequent cause of inherited hearing loss worldwide and in Iran after *GJB2* mutations.^2^ The mutation spectrum of these two genes is ethnic-specific and varies among different populations.^2^ Until now, about 704 mutations have been recorded in *SLC26A4* in the Human Gene Mutation Database (HGMD).^3^ *SLC26A4* (Solute Carrier Family 26, Member 4) is a known Hearing Loss (HL) gene with 21 exons, located on 7q22.3 which encodes pendrin, an iodide–chloride transporter expressed in the thyroid, inner ear, and kidney.^4,5^ Homozygous or compound heterozygous mutations in *SLC26A4* are associated with a broad phenotypic spectrum, from Pendred syndrome; PDS (MIM: 274600) to Deafness, autosomal recessive 4 (DFNB4), with enlarged vestibular aqueduct (EVA); DFNB4 (MIM: 600791).^4^ PDS, the most common form of syndromic hearing loss, is characterized by congenital sensorineural hearing loss, thyroid dysfunction, and severe-to-profound temporal bone abnormalities. Unlike PDS, DFNB4 with an EVA is recognized in patients without thyroid dysfunction or other systemic features.^6,7^ *SLC26A4* mutations are common in different countries and regions, and some are ethnic-specific.^8^ Elucidating the distribution of *SLC26A4* pathogenic variants in various regions of our country is of paramount importance for designing mutation-screening programs throughout the country for patients with HL, particularly those with goiter or enlarged vestibular aqueduct symptoms.^8,9^ To date, various* SLC26A4* mutations with different frequencies have been observed in different regions of Iran.^1,10^ Of these, c.965dup (p.Asn322LysfsTer8) is a founder mutation originating from northwestern Iran, which further supports the ethnic and regional differences in the* SLC26A4* mutation spectrum.^1,4^ Recently, we widely observed the known (c.716T > A; p.Val239Asp) mutation in southeastern Iran near the border of Pakistan, where the founder effect of this variant is assumed.^11^ Our finding in this report, in addition to unraveling the molecular cause of NSHL in two unrelated consanguineous Baluch families, provide further insight into the genetic background of this region, and may also be more proof of the founder effect of the p.Val239Asp mutation in Pakistan and the footprint of probable common ancestry and migration story.

## Case Report

**Figure 1:**
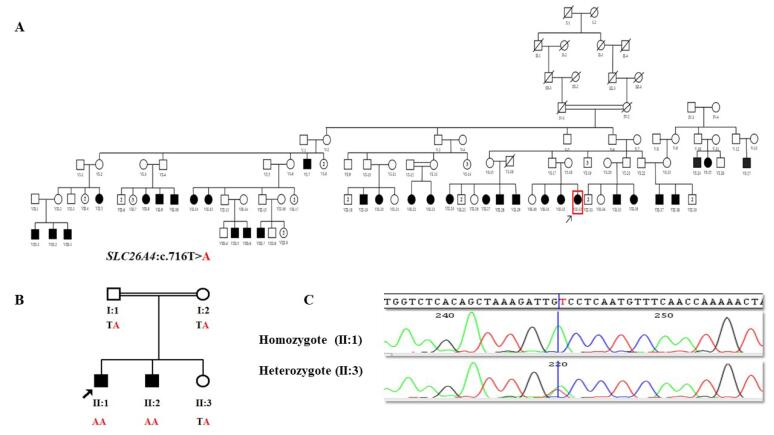
** A) **Pedigree of family 1,** B) **Pedigree of family 2,** C) **An electropherogram of the c.716T > A mutation in one homozygote affected and one heterozygote healthy member of family 2

 We identified two unrelated consanguineous Baluch families in southeastern Iran, one of which was large and multi-branched with 31 HL patients and the other with 2 affected members (Figure 1). Prior to sample collection, informed consent was obtained by the ethical committee of the USWR from each family member who agreed to participate in the study. A complete physical examination did not reveal any syndromic features and showed normal thyroid function and vision. The tympanometry test and otoscopic examination of each family indicated that the patients had severe to profound hearing loss. Eventually, the proband of each family, whose genetic screening was negative for *GJB2* pathogenic variants, underwent Exome Sequencing (ES). Illumina NextSeq500 (Illumina, San Diego, California, USA) and Agilent SureSelectXT Human All Exon V6 (Agilent Technologies Inc., Santa Clara, CA, USA) were used for ES. Sequences were mapped to the UCSC hg38 human reference genome using the Burrows-Wheeler Aligner (BWA).^12^ VCF files were generated using the Genome Analysis Tool Kit (GATK)^13^ and annotation was performed using ANNOVAR.^14^ Variants were filtered based on their quality/coverage depth ( ≥ 3) and minor allele frequency (MAF < 0.5%). Variant prioritization was performed by considering the types of variants and bioinformatics predictions. All potentially causing variants detected by ES were validated by Sanger sequencing using an ABI 3500 Sequencer, and co-segregation studies were performed, including all available and informative family members. The primers used in this study for sequencing the candidate variants included the forward 5’-AGGTTTCTATCTCAGGCAAACA-3’ and reverse 5’-GCCCAGACTCAGAGAATGAA-3.’

 By analyzing the ES data of each proband of these two families, we interestingly detected the same known pathogenic missense variant, *SLC26A4* (NM_000441.2):c.716T > A (p.Val239Asp), in both families (Table 1). Sanger sequencing confirmed the presence of this pathogenic variant in their remaining affected members (Figure 1).

**Table 1:** Description of the Causative Variant in These Patients

**Gene**	**Transcript**	**Variant**	**Exon**	**SIFT**	**REVEL**	**CADD PHRED Score**	**ACMG Classification**
*SLC26A4*	NM_000441.2	chr7:107675060:T > A c.716T > A (p.Val239Asp)	6	Pathogenic supporting (0.001)	Pathogenic moderate (0.935)	27.0	Pathogenic

## Discussion

 The total prevalence of *GJB2-*dependent HL in Iran is 16.5%; however, it varies in different regions of Iran from 38.3% to zero in the North and South, respectively.^1^ Regardless of *GJB2*, *SLC26A4* mutations, with a frequency of 16.25%, are the highest contributors to NSHL in the Iranian population.^1^

**Figure 2:**
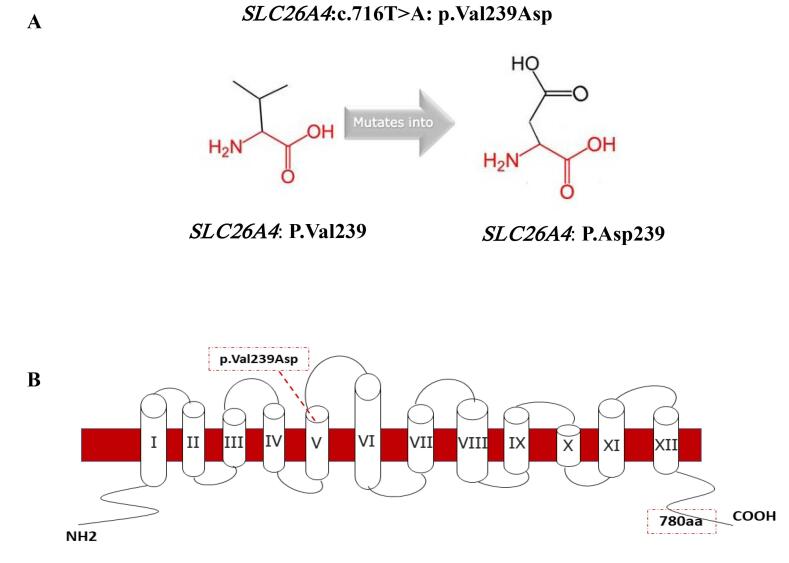
** A) **Schematic Structures of the Original (left) and the Mutant (right) Amino Acid for the Substitution of Valine Into Aspartic (https://www3.cmbi.umcn.nl/hope/),** B) **The p.Val239Asp Mutation in the Schematic Model of the Human Pendrin Protein

 In this study, we investigated the genetic cause of NSHL in two unrelated consanguineous Baluch families from the Sistan and Baluchestan province and identified *SLC26A4* (NM_000441.2): c.716T > A (p.Val239Asp) mutation as a cause of HL in these families. This transversion mutation in exon 6 of *SLC26A4* leads to the substitution of Valine into Aspartic Acid at position 239 of the pendrin protein which comprises 780 amino acids and contains 12 transmembrane domains. As this residue is located in the core of the transmembrane domain, the difference between the wild-type and mutant residues can disrupt the structure of this domain (Figure 2).^15,16^

**Figure 3:**
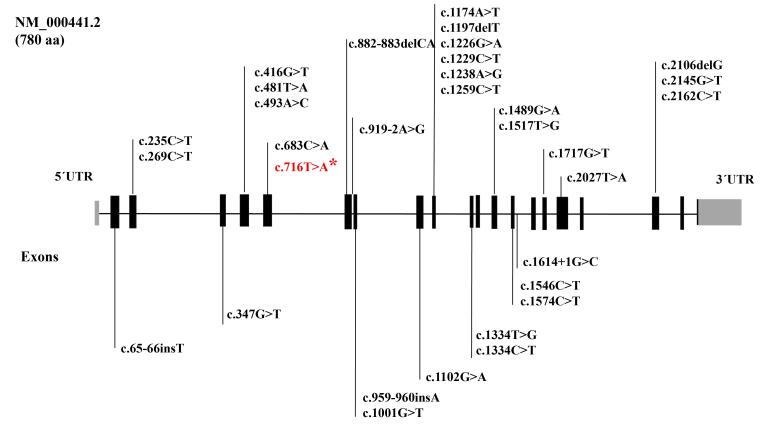
*SLC26A4* Mutations Responsible for ARNSHL in Iranian Families Until 2022. * The mutation identified in patients in this study

**Figure 4:**
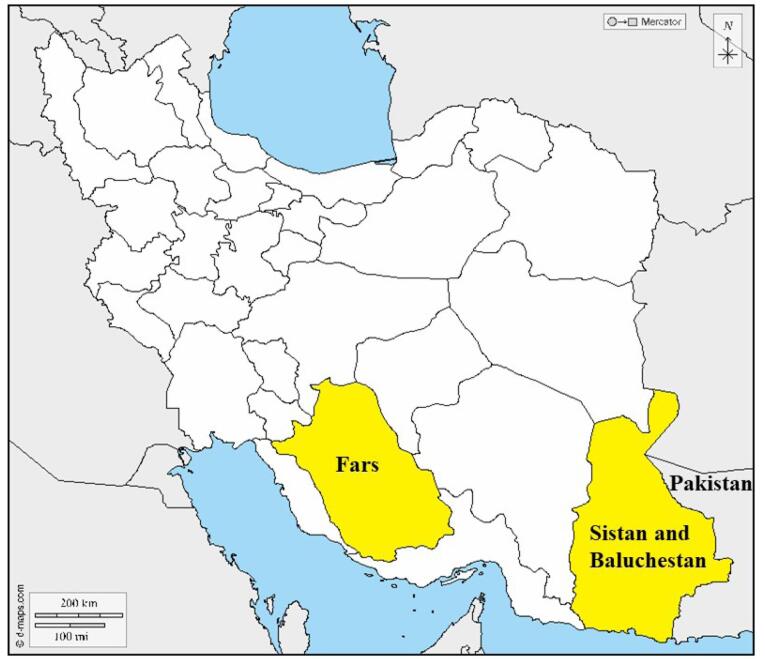
Geographic Distribution of (c.716T > A; p.Val239Asp) in Our HL Cohort

 The results of this study, especially a large multi-branch family with 31 affected members from a small village in the border areas of Iran, prompted us to conduct further research on the origin and distribution of *SLC26A4* and p.Val239Asp. So far, various mutations of *SLC26A4* have been reported in Iran, which include a wide range of phenotypes from “Pendred syndrome” to non-syndromic “Deafness, autosomal recessive 4, with enlarged vestibular aqueduct”. Figure 3 shows various *SLC26A4* mutations responsible for autosomal recessive non-syndromic hearing loss (ARNSHL) in Iranian families until 2022.^1^ A detailed investigation of our cohort data revealed that in addition to the two families mentioned in this paper, we previously identified five other families harboring this mutation. Among the 48 affected members of the seven families investigated in our cohort, 38 were Baluch (Figure 4, Table 2).^1,17^ In a review of studies on this mutation by other groups in Iran, only one family was reported with three affected members from the Kurdistan province with Kurdish ethnicity.^18^

**Table 2:** Ethnicity and Number of Affected Harboring (c.716T > A; p.Val239Asp) in our HLCohort^1^

**#No**	**Family**	**Number of Affected in Pedigree**	**Variant**	**Ethnicity**
1	Family 1	31	*SLC26A4*(NM_000441.2):c.716T > A (p.Val239Asp)	Baluchis
2	Family 2	2		Baluchis
3	Family 3	4		Baluchis
4	Family 4	1		Baluchis
5	Family 5	3		Persians
6	Family 6	5		Persians
7	Family 7	2		Persians

 In 2015, Tsukada et al reviewed the origin and spectrum of *SLC26A4* mutations worldwide and showed that p.Val239Asp is the most common mutation in Pakistan (35.6%) and Turkey (33.3%).^2^ Several studies have proposed a founder effect of p.Val239Asp in Pakistan.^11,19,20^ Finding the p.Val239Asp mutation in a large number of patients in the Sistan and Baluchestan province, which lies on the border with Pakistan, might reflect the magnification of the founder effect and contribute to the final genetic makeup of this region’s population.

## Conclusion

 The results of this study suggest that the gene flow of the p.Val239Asp mutation from Pakistan, a country neighboring southeastern Iran, likely changed the frequency of this mutation in that region. Given the high prevalence and mutation-specific origin of *SLC26A4* mutations, considering the ethnic and regional backgrounds of patients is helpful in genetic counseling and clinical decision-making, and evaluating *SLC26A4* mutations can be a first-tier screening in regions with high prevalence.
